# Wool: From Properties and Structure to Genetic Insights and Sheep Improvement Strategies

**DOI:** 10.3390/ani15192790

**Published:** 2025-09-25

**Authors:** Huitong Zhou, Lingrong Bai, Shaobin Li, Jiqing Wang, Jon G. H. Hickford

**Affiliations:** 1 International Wool Research Institute, Faculty of Animal Science and Technology, Gansu Agricultural University, Lanzhou 730070, China; huitong.zhou@lincoln.ac.nz (H.Z.); lisb@gsau.edu.cn (S.L.); wangjq@gsau.edu.cn (J.W.); 2 Gene-Marker Laboratory, Faculty of Agriculture and Life Sciences, Lincoln University, Lincoln 7647, New Zealand; lingrong.bai@lincolnuni.ac.nz

**Keywords:** wool fibre, keratin, keratin-associated protein, molecular genetics, gene polymorphism, genetic association, genetic improvement, sheep, fibre trait, breeding, wool quality

## Abstract

The wool of sheep consists of structurally intricate natural fibres that can be processed and manufactured into a range of products. It is prized for its insulation, moisture-buffering capability, flame resistance, and biodegradability. These features arise from its unique fibre architecture and specialised protein composition, which set it apart from most other natural and synthetic fibres. However, despite these novel characteristics, wool fibre variation hampers its uses and reduces its ability to compete with other fibres. This review summarises our current knowledge of wool fibre biology. It begins with a description of wool’s functional properties and performance attributes, then explores the structural foundations of these properties, the molecular basis of fibre trait variation, and prospects for improving fibre quality using genetic approaches. Particular attention is given to the wool keratin and keratin-associated protein genes, their spatiotemporal expression patterns, and genetic polymorphism that may influence fibre characteristics. Opportunities for the genetic improvement of sheep are discussed, including the use of genetic modification and marker-assisted selection. Challenges in interpreting gene–trait associations, particularly from high-throughput omics studies, are highlighted, along with the need for functionally validated genetic markers. Potential trade-offs between wool characteristics and other production and reproductive traits are considered, emphasising the need for balanced breeding approaches. By integrating insights from structural biology, molecular genetics, and breeding strategies, this review provides a foundation for wool fibre improvement.

## Simple Summary

The wool of sheep provides a fibre with exceptional properties. This makes it ideally suited for the manufacture of performance textiles, including ‘next-to-skin’ applications. This review provides an overview of wool and its biology, with a focus on the molecular genetics that underpin fibre traits and how that may allow for the genetic improvement of sheep to produce better wool. It will highlight the roles of keratin and keratin-associated protein genes in producing wool fibres, how they are genetically variable, their expression patterns, and how the genetic variation may affect wool fibre traits. The review also explores how genetic tools could be used to enhance wool quality while considering possible trade-offs with other traits. The integration of phenotypic, molecular, and genetic knowledge will be essential for future advancement in wool production and the improvement of fibre quality.

## 1. Introduction

Environmental concerns and the demand for sustainable materials have renewed scientific and industrial interest in natural fibres. Among these fibres, the wool of sheep stands out for its functional properties that other natural and synthetic fibres have difficulty emulating. These include its insulative properties, flame resistance, resilience, and hygroscopic properties [1]. Wool can be used to manufacture environmentally responsible fibre products like clothing, and these can also be biodegradable or recyclable.

Wool is a proteinaceous fibre that is shorn from sheep, albeit not all breeds are considered to be wool producers. In this context, sheep numbers are increasing globally [2], with the International Wool Textile Organisation (IWTO) reporting that sheep numbers rose to 1.296 billion heads in 2022, with greasy wool production rising to 1977.3 million kilogrammes, and clean wool production reaching an estimated 1051.2 million kilogrammes in that year.

Despite wool’s long history of use, many of its functional properties remain under-recognised, and their biological basis is not fully understood. While much of the physical structure of wool fibres has been described [3], the molecular mechanisms and environmental factors responsible for creating variation in fibre traits are only partially understood. Accordingly, a challenge remains to improve wool quality and achieve greater fibre uniformity, which is necessary if the performance and value of wool are to be further enhanced.

This review summarises our knowledge of wool’s functional properties and performance attributes and explores the structural and molecular bases of fibre traits. Particular attention is given to wool keratin and keratin-associated protein genes, their expression patterns, and genetic variation that may influence fibre characteristics. Opportunities for genetic improvement, through genetic modification and selective breeding, are also discussed. Challenges in interpreting gene–trait associations, the need for validated genetic markers, and potential trade-offs between wool characteristics and other production and reproductive traits are considered. By integrating insights from structural biology, molecular genetics, and breeding strategies, this review provides a comprehensive foundation for improving wool fibres.

## 2. Key Functional Properties of Wool Fibre

Wool possesses a range of functional properties that contribute to its performance across various practical applications. Although other fibres may surpass wool in individual aspects of performance, it has a combination of functional properties that other fibres have difficulty matching.

### 2.1. Thermal and Acoustic Insulation

Wool fibres possess a naturally crimped structure. When spun into yarn or felted, they can be used to create products containing air pockets that trap air and provide an insulative layer. These pockets can reduce heat transfer, which in clothing helps to stabilise body temperature and enhance thermal comfort. As an insulative layer, the thermal conductivity of wool typically ranges from 0.038 to 0.045 W/m·K, which is comparable to conventional insulation materials such as mineral wool and expanded polystyrene products [4]. The variation in conductivity reflects variation in the wool fibres and the way in which they are manufactured into clothing, with characteristics like the degree of medulation, mean fibre diameter, and curvature, affecting thermal conductivity.

The crimped fibre structure can also contribute to acoustic insulation. As sound waves pass through wool products, they are gradually dispersed and absorbed due to friction with air and fibre surfaces. This reduces sound intensity and reverberation. Wool-based materials can demonstrate strong sound absorption coefficients, particularly in the mid-frequency range of 500 to 2000 Hz [5], albeit this study recognises that variation in the thickness of the wool material tested affects this property. Accordingly, fibre traits like curvature and fibre diameter are likely to affect the ability to absorb sound. This makes wool effective for mitigating everyday noise in both residential and commercial environments.

### 2.2. Moisture Regulation

Wool is hygroscopic and has the ability to absorb and release moisture without feeling damp. It has a water retention capacity of up to approximately one-third of its weight [1]. This capability distinguishes wool from many other natural and synthetic fibres and is fundamental to its thermal ‘comfort’ when used in clothing.

Specifically, when water molecules bind to wool fibres, they undergo an exothermic process that produces heat (described as ‘heat of sorption’). Conversely, in warmer conditions (as may occur when woollen clothing is worn during physical activity), the wool fibres release absorbed water via evaporation. This is an endothermic process that cools the body by drawing heat away. Wool’s heat of sorption is high; estimated at 100–115 J/g for dry fibres [1], albeit this will vary with wool type [6].

This ‘bidirectional’ moisture management property (both absorption and desorption) provides wool clothing with superior thermoregulatory characteristics when compared to other fibre choices, and across a range of climatic conditions. In comparison, cotton, while also hygroscopic, absorbs less moisture and only has a moderate heat of sorption (~46 J/g) [7]. This can cause cotton clothing to have a clammy feeling when wet, and it provides far less thermal benefit. Synthetic fibres such as polyester and acrylic absorb little water, with minimal heat of sorption (about 5–7 J/g) [7]. Accordingly, many synthetic fibres rely primarily on moisture wicking, rather than absorption, and thus they offer limited thermal buffering, albeit reasonable insulative properties.

### 2.3. Chemical and Odour Absorption

Wool is effective at absorbing and neutralising various volatile organic compounds (VOCs) and odours. It can bind airborne pollutants such as formaldehyde, sulphur dioxide, and nitrogen oxides (NOx) through chemisorption, forming stable compounds that enable long-term pollutant removal [8]. For example, wool carpets can reduce formaldehyde levels from 300 ppm to near zero within hours [9], and with many modern building products (e.g., pressed-wood products, particle boards, plywood, paint, glue, and foam insulating products) releasing formaldehyde through a process called off-gassing, this property is helpful for removing a known human carcinogen from buildings.

In clothing, wool can reduce body odour by binding the molecules responsible for those smells. Wool garments, including socks and activewear, typically develop less odour than synthetic materials, even after prolonged wear, enhancing freshness and wearer comfort [3].

Wool’s natural oleophilic properties allow it to absorb oil up to 40 times its own weight, depending on the oil’s viscosity [10]. This makes wool well suited for oil spill clean-up in contaminated environments, and when blended with synthetic fibres to increase its buoyancy, it can be used to recover oil from water [3].

Wool also exhibits an affinity for absorbing metals like mercury, gold, and lead [11]. Powdered wool, with increased surface area, has increased uptake rates, making it effective for removing heavy metals from industrial effluents and contaminated water. This property is useful for filtration applications, including the treatment of mercury-contaminated effluents from the chlor-alkali industry [12]. There is a suggestion that wool is an effective filter absorbent for microplastic removal from water [13].

### 2.4. Fire Resistance and Safety

Wool has a high limiting oxygen index (LOI) of approximately 25.2%, albeit this will vary depending on the fibre density of the materials and the presence of moisture (given wool’s hygroscopic nature). This is above the oxygen concentration in air (~21%), meaning that wool is more flame-resistant than fibres such as cotton (LOI ~18.4%), nylon (~20.1%), and polyester (~20.6%) [14]. In this context, the fibre has been used for fire protection for many years, with the London Fire Brigade describing its use in woollen tunics over the period 1866–1989, until the first Nomex (a synthetic meta-aramid fibre) suits were introduced [15].

Wool also has an ignition temperature that ranges from 570 °C to 600 °C. In comparison, cotton ignites at a much lower temperature (~255 °C). Synthetic fibres like nylon and polyester ignite between 485 and 575 °C [14], but have lower melting temperatures that can lead to structural failure in products manufactured from them. The high ignition threshold of wool reduces the likelihood of it igniting when exposed to heat sources, and if ignited, wool does not melt but instead chars. This forms a protective thermal barrier that suppresses flame spread and shields underlying materials. In contrast, if synthetic fibres melt and drip, they can increase the risk of secondary burns, and the ignition of synthetic fibres can produce carbon monoxide, dioxins, hydrogen cyanide, and nitrous oxides.

When burning, wool has a lower heat of combustion (~20.5 kJ/g, compared to 22.6 kJ/g for nylon), meaning it releases less energy compared to other fibres [14]. This limits flame intensity and reduces the potential for fire propagation, further enhancing its fire safety profile.

Taken together, these properties make wool a safer choice in contexts where fire safety is critical. Its unique combination of high ignition temperature, low heat of combustion, and non-melting behaviour provides a level of protection not commonly found for other natural or synthetic fibres. As a result, wool can be used in applications for protective clothing, mattress covers, upholstery, and interior textiles, where reducing fire risk is important.

### 2.5. UV Shielding

Wool fibres offer effective protection against ultraviolet (UV) radiation, making them well-suited for use in outdoor garments, coverings, and applications where UV shielding is important. A study by Gambichler et al. [16] that assessed 236 spring and summer apparel fabrics revealed that wool outperformed common fabrics such as cotton, linen, viscose, and polyester in UV protection. Over 70% of wool samples achieved a UPF rating of 50+, with even the worst-performing wool fabric reaching a UPF of 40+. Wool’s high UV protection is considered an advantage and is attributed to its unique fibre structure and its ability to absorb and scatter UV radiation.

### 2.6. Durability, Elasticity and Resilience

Wool is valued for its ability to maintain the appearance, fit, and performance of garments. It offers excellent durability, withstanding repeated wear, bending, and handling without a major loss of performance or comfort.

Wool fibres are elastic and capable of stretching up to 30% when dry and 50% when wet, while remaining capable of returning to their original shape [17]. This attribute helps garments maintain their fit or drape, enhances comfort, and contributes to wrinkle resistance by allowing the fibre to recover its form after deformation. Wool also displays outstanding resilience to repeated bending and is capable of enduring up to 20,000 bends without breaking. This is more than natural fibres such as cotton (∼3000 bends) and silk (∼2000 bends) [17]. This resilience reduces the likelihood of fibre breakage under repeated stress, thereby supporting long-term performance and durability.

Wool fibres are abrasion resistant, mainly due to their tough exterior layer of scales, which helps protect them from surface damage. This makes wool suitable for high-friction applications such as may be needed for carpets, upholstery, and outerwear. It recovers shape well after compression, especially in humid conditions, which helps maintain shape and appearance over time.

Together, these properties enable wool products to remain durable, comfortable, and visually appealing even after repeated use and care. As a result, wool is a reliable choice for both everyday wear and demanding applications where longevity and consistent performance are essential.

## 3. Health and Environmental Benefits of Wool

Beyond its functional performance, wool offers benefits for human health and wellbeing. It also has good environmental credentials.

### 3.1. Health and Wellbeing

Wool possesses a variety of properties that contribute to human health, comfort, and safety. Its breathability, ability to manage moisture, and softness make it suitable for next-to-skin use. Other properties help improve indoor environmental quality and reduce health risks. Together, these attributes support wellbeing across diverse everyday and specialised environments.

#### 3.1.1. Skin Health

Contrary to traditional views that wool irritates sensitive skin, clinical studies show that superfine Merino wool (defined as fibres finer than 18.5 µm, ideally <16.5 µm for sensitive skin), is not only well tolerated but may even benefit individuals with atopic dermatitis (AD), a common chronic inflammatory skin condition.

In a randomised controlled trial, Su et al. [18] revealed that children with mild-to-moderate AD experienced a reduction in disease severity (as measured by scoring atopic dermatitis [SCORAD] scores) after wearing superfine Merino wool garments for 3 and 6 weeks compared to cotton. Participants also reported less reliance on corticosteroids and improved comfort and quality of life. Interestingly, when participants returned to cotton clothing, their symptoms worsened, highlighting the therapeutic potential of superfine Merino wool. Spelman et al. [19] extended the evaluation period to 15 weeks and revealed that children and young adults with AD who wore superfine Merino wool base-layer garments experienced improvements across multiple validated AD severity scales, including SCORAD, eczema area, and severity index (EASI). No negative effects or symptom worsening were observed. Participants also reported enhanced comfort and garment practicality. Similarly, Fowler et al. [20] reported improvements in eczema severity (EASI scores), dermatology-related quality of life, and clinical assessments in patients with AD wearing Merino wool for 6 weeks, with no adverse skin reactions reported.

Complementing these clinical trials, Zallmann et al. [21] observed no evidence that wool causes allergic contact dermatitis. Instead, most skin reactions to wool are non-immune in nature, typically caused by mechanical irritation from coarse fibres rather than any allergenic effect. Taken together, these clinical and dermatological findings strongly support the safety and potential benefits of superfine Merino wool for individuals with sensitive skin or AD. They challenge outdated perceptions of wool intolerance and reaffirm its suitability for next-to-skin applications.

#### 3.1.2. Sleep Quality

Emerging evidence supports wool’s role in enhancing sleep quality, particularly when used in next-to-skin applications such as sleepwear. Clinical studies using polysomnography have demonstrated that Merino wool sleepwear can positively influence various aspects of sleep. In a controlled study with young healthy adults (aged 20–31 years), Shin et al. [22] revealed that at a lower ambient temperature (17 °C), wool sleepwear reduced sleep-onset latency compared to cotton. The trial participants spent more time in deep sleep (N3 stage) than at a higher temperature (22 °C). These benefits are attributed to wool’s ability to buffer humidity and insulate against temperature fluctuations, helping to create a dry, thermo-neutral microenvironment. No effects were observed for woollen bedding material, suggesting that sleepwear, due to its direct skin contact, has a more pronounced influence on sleep-related thermal comfort.

Similar benefits were observed in other adults (aged 50–70 years) and individuals with poor sleep quality. Chow et al. [23] reported that under warm conditions wool sleepwear resulted in shorter sleep-onset latency, less sleep fragmentation, and reduced wakefulness compared to cotton and polyester. Wool sleepwear promoted improved sleep, particularly for those aged 65 years and older, and for poor sleepers in warm ambient conditions.

A recent review by Li et al. [24] examined studies comparing fibre types in sleepwear and bedding and reported that wool sleepwear improved sleep onset in adults in cool conditions and in older adults in warm conditions. While study designs varied in temperature conditions, fibre types, and participant groups, the review highlights wool’s potential to support better sleep through its thermal and moisture buffering properties. Together, these findings suggest that wool sleepwear contributes to improved sleep quality, especially in cooler or more challenging environments, and among individuals with heightened sleep sensitivity.

### 3.2. Environmental Sustainability

In addition to its contributions to human health and wellbeing, wool also offers a range of environmental advantages that reinforce its role as a fibre that is both renewable and biodegradable. Sheep continuously grow fleece, which is typically shorn off once a year. This production relies on chemical energy derived from photosynthesis that is stored in plant biomass. Sheep convert this biomass into wool, which contributes to biogenic carbon sequestration, and it offers short- to medium-term carbon storage that helps offset emissions associated with production. In this respect, one industry report states that approximately 50% of the dry weight of wool is carbon, captured from atmospheric CO_2_ through photosynthesis and stored in the fibre as sheep consume pasture [25], and that each kilogram of clean wool stores approximately 1.8 kg of CO_2_ equivalents.

This is, however, a simplification of a much more complex situation wherein multiple factors affect carbon sequestration and greenhouse gas (GHG) release, including, but not limited to, the variation in farming systems, sheep breed effects, allocation of the emission footprint between meat, hides, lanolin, milk, and wool production, variation in data from different regions, and variation in assessment methodology. This is discussed in some detail by the International Wool Textile Organisation (IWTO; Brussels, Belgium), the global authority for standards in the wool textile industry, and in recent research by Wiedemann et al. [26] which highlights the challenge in undertaking a life cycle assessment (LCA) for a woollen product.

While methane from sheep is a potent GHG, there is debate around the use of different metrics for assessing climate impacts for what is considered to be a short-lived climate pollutant (unlike CO_2_), and with a recent suggestion that a new metric called GWP* should be used instead of the current standard Global Warming Potential (GWP) [27].

Biodegradability is another environmental advantage of wool. Composed primarily of proteins, the fibres break down in terrestrial environments, typically decomposing within a few months in soil [28], although this depends on chemical processes applied during manufacturing, including dyeing, bleaching, stain-repellence, and various shrink-resist treatments. During this breakdown, wool releases nitrogen, sulphur, phosphorus, and carbon back into the ecosystem. In contrast, synthetic fibres can persist in the environment for centuries, with this contributing to plastic pollution and ecological harm. Unlike synthetic textiles such as polyester or acrylic, wool textiles biodegrade rapidly, and the fibre does not directly contribute to microplastic pollution in aquatic environments [29]. This makes it a safer choice for both terrestrial and aquatic environments, albeit the processing of wool fibre and manufacture of wool products can involve steps that can have negative environmental impacts.

This natural biodegradation creates a closed-loop biological cycle: plants capture solar energy and atmospheric CO_2_ to produce biomass, which sheep consume and convert into wool. When wool is discarded, it biodegrades in the soil, releasing carbon, nitrogen, sulphur, and phosphorus that can resupply minerals to growing plants. This creates a biologically based circular or regenerative economy.

In addition, wool’s durability and longevity further strengthen its environmental credentials. Wool garments tend to be worn longer, need washing less frequently, and maintain their performance and appearance better than many fast fashion materials. Wool garments can often be refreshed by airing rather than washing due to the fibre’s natural soil repellence and odour inhibiting properties [30], and washing is typically undertaken at lower temperatures and using shorter and gentler washing cycles. These qualities reduce the need for frequent replacement and lower environmental impact.

Together, these factors position wool as one of the more environmentally responsible fibres available, aligning with values underpinning regenerative agriculture, circular material flows, and climate-conscious design. Despite these advantages, it does face competition from synthetic fibres, which currently dominate global textile production due to their low cost, versatility, uniformity, strength, elasticity and ease of large-scale manufacturing. However, this dominance comes with environmental costs, including reliance on non-renewable petroleum resources, high energy inputs, high water footprints and microplastic pollution. This highlights wool’s potential as a more sustainable alternative.

## 4. Structural Basis of Wool’s Functional Properties

The functional properties of wool fibres arise from their intricate structure, which has been described and illustrated in detail elsewhere, for example, by Caven et al. [31] and Rippon [32]. This structure confers multifunctionality that is difficult to replicate with synthetic fibres. Understanding this structure is essential to appreciating the diverse performance advantages that wool fibres offer.

### 4.1. The Cuticle: The Surface Structure and Protective Outer Layer

**Figure 1:**
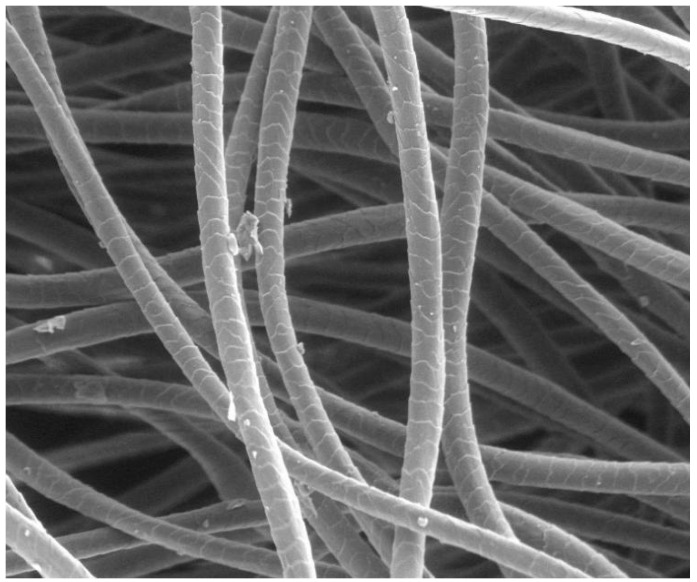
Electron microscopic image of scoured Merino wool fibres with a mean fibre diameter of 19.5 µm, showing the over-lapping cuticle scales.

The outermost layer of wool fibres is the cuticle, a sheath of overlapping, scale-like cells that constitute approximately 10% of the fibre’s mass. These scales overlap in the longitudinal direction like tiles on a roof, with their edges pointing from the fibre root to its tip [32,33] (Figure 1).

The cuticle is composed of two major layers: the outer exocuticle and the inner endocuticle. In Merino wool, the exocuticle is approximately 0.3 µm thick, accounting for about 60% of the cuticle volume. It includes a dense outer region known as the A-layer and contains a substantial portion of the cystine present in the cuticle. The endocuticle of Merino wool, approximately 0.2 µm thick and making up about 40% of the whole cuticle, has a lower cystine content and is considered more amorphous and susceptible to chemical or enzymatic attack [32]. Surrounding each cuticle cell is the epicuticle, a thin (2–7 nm thick) proteinaceous membrane covalently bound to a monolayer of lipids, predominantly 18-methyleicosanoic acid (18-MEA; a branched-chain fatty acid). This lipid layer is approximately 0.9 nm thick and plays a key role in making wool fibres water- and dirt-resistant [32].

The cuticle structure of wool fibres varies with fibre diameter. In fine wool fibres, such as those of Merino sheep, the cuticle is typically a single cell layer thick. This single-layer arrangement primarily envelops the orthocortex, but in regions adjacent to the paracortical cells, two to three cuticle layers can be observed. In contrast, coarse wool fibres possess a thicker cuticle, which can comprise up to 15 layers of cells [23]. This increased cuticle thickness in coarse wool contributes to greater fibre rigidity and enhances resistance to external factors, but reduces fibre flexibility and softness.

These structural characteristics underpin several key functional properties of wool [32,33]. The overlapping scale arrangement creates directional friction, enabling felting and self-cleaning properties. The gaps between these scales, despite the hydrophobic surface, allow water molecules to diffuse into and out of the fibre, facilitating moisture movement. Additionally, the multi-layered cuticle and high cysteine content enhance abrasion resistance, with this contributing to the fibre’s mechanical durability.

### 4.2. The Cortex: Internal Architecture and Contribution to Fibre Performance

Beneath the cuticle lies the cortex, which makes up approximately 90% of the fibre’s volume and features a sophisticated composite architecture. The cortex is primarily composed of two types of cortical cells, orthocortical and paracortical, with intermediate mesocortical cells occasionally present [32].

In fine wool fibres, such as those of Merino sheep, these cortical cells are arranged side-by-side in semi-cylindrical arrangements that twist around each other along the fibre length [32]. In contrast, coarser wool fibres, such as those from Lincoln sheep, tend to exhibit a concentric arrangement of cortical cells, with orthocortical cells typically located centrally. Some coarse animal hairs contain only orthocortical cells, while others, particularly in very coarse fibres, contain a central medulla, an air-filled cavity that enhances the fibre’s insulation properties [32]. Medullation is often observed in wool fibres of larger diameter.

While the mechanisms responsible for crimp formation remain incompletely understood, one plausible explanation is that crimp arises primarily from the bilateral arrangement of ortho- and paracortical cells, combined with differences in their physical and chemical properties. These differences, such as unequal expansion during keratinisation [34], variations in relative longitudinal cell lengths [35], and distinct responses to moisture, can generate internal stresses between the two cell types. When arranged bilaterally rather than concentrically, these stresses are asymmetrically distributed across the fibre cross-section, potentially leading to bending along the fibre axis. This process may give rise to the characteristic three-dimensional curvature observed as crimp, with the orthocortex typically oriented toward the outside of each crimp wave and the two cortical segments twisting along the fibre in phase with the crimp pattern [32].

This crimp structure plays a critical role in wool’s functional performance. It introduces bulk, compressional resilience, and elasticity, which enhance comfort and durability in textile applications. The repeating waves of crimp create spaces between fibres, trapping air and contributing to thermal and acoustic insulation by reducing the transfer of heat and sound.

### 4.3. Microstructural Organisation Within Cortical Cells

Within cortical cells, larger structural units known as macrofibrils are aligned longitudinally along the fibre axis and can be further subdivided into finer microfibrils. In the orthocortical cells the macrofibrils are densely packed and arranged in a helical pattern, whereas in the paracortical cells, they tend to be more loosely organised and exhibit a pseudo-hexagonal packing aligned parallel to the fibre axis [32].

The microfibrils are composed of α-helical keratin intermediate filaments (KIFs), each formed by coiled-coil heterodimers of type I and type II keratin polypeptide chains. These wool keratins are orthologous to the ‘hair keratins’ of humans and other mammals, but because they are specifically expressed in wool fibres, they are referred to here as ‘wool keratins’, to distinguish them from the keratins expressed in other epithelial tissues [36]. Two keratin heterodimers align in an antiparallel arrangement to form a four-chain unit, which further assembles into larger rod-like intermediate filaments with a telescopic configuration [32]. These structures provide tensile strength and internal cohesion, enabling the fibre to withstand stretching and mechanical stress.

Surrounding the KIFs is a matrix composed of keratin-associated proteins (KAPs), including high-sulphur (HS), ultra-high sulphur (UHS), and high-glycine/tyrosine (HGT) KAPs. These proteins interact with each other and with KIFs through covalent and non-covalent mechanisms, including disulphide and isopeptide bonds, hydrogen bonding, hydrophobic interactions, and ionic forces [32]. This proteinaceous KAP matrix fills the space between KIFs, contributing to the cohesion and plasticity of the fibre and modulating its mechanical properties. Covalent cross-links enhance strength and durability, while non-covalent interactions support flexibility and energy dissipation. This collectively contributes to wool’s resilience and its ability to recover after deformation.

The organisational differences between the ortho- and paracortical cells are accompanied by variations in the protein composition. A key difference lies in the relative abundance of KIFs and KAPs. Orthocortical cells contain a higher ratio of KIFs to matrix, while paracortical cells have a greater proportion of matrix [37]. Differences in KAP group profiles between these two cell types have also been reported, with HGT-KAPs more prevalent in orthocortical cells and UHS-KAPs more abundant in paracortical cells, but this pattern is not consistently observed across studies [38]. However, because only a limited subset of the KAPs were characterised at the time of these studies, the trends should be regarded as suggestive and thus be interpreted with caution.

These microstructural and biochemical differences play a vital role in defining the physical properties of the fibre. The configuration and interaction of KIFs and KAPs influences mechanical properties such as strength, extensibility, and resilience. Wool proteins contain polar and charged amino acid residues which facilitate hydrogen bonding and ionic interaction with water molecules [1]. This amphoteric nature underpins wool’s high moisture regain. The amorphous regions within the matrix accommodate water and small molecules without compromising structural integrity, while the flexible protein network allows for dynamic moisture uptake and release. Together, these contribute to wool’s effective buffering of temperature and humidity.

Wool proteins are rich in amino acids bearing functional groups capable of interacting with a wide range of substances. In particular, cysteine, aspartic acid, glutamic acid, serine, and threonine are present in high abundance, while tyrosine is moderately abundant (3.9 mol%) [39]. The thiol group in cysteine, the carboxyl group in aspartic and glutamic acids, the hydroxyl group in serine, threonine, and tyrosine, along with the aromatic ring in tyrosine together enable wool to bind a range of substances, including odorous molecules, VOCs, and heavy metals. This molecular composition underpins wool’s capacity to sequester environmental pollutants and odours. The high nitrogen (approximately 16 wt%) and sulphur (approximately 5 wt%) content of wool proteins [40] contributes to its inherent flame retardancy by promoting char formation and suppressing melting and dripping during combustion.

Altogether, this sophisticated structural design bestows wool fibre with a range of novel performance properties. It also underpins many of wool’s unique health-related and wellbeing benefits. These attributes contribute to making wool fibre a remarkable material, one that rightfully earns its title as ‘nature’s wonder fibre’ [41].

## 5. Molecular Genetics of Wool Fibre Traits

Despite its functional value, wool suffers from variability in fibre traits including diameter, curvature, length, and strength. This phenotypic variation reduces fibre uniformity, complicates processing, and affects the commercial value of wool. These traits arise from intricate and hierarchical molecular processes during wool follicle development and fibre growth.

Various genes and metabolic systems have been revealed to affect wool growth. These systems can be separated into those that operate prior to, or during wool follicle activity, and those that are directly involved in producing the structural components of the fibre. Genes (and the pathways to which they contribute) of interest in the former group include, but are not limited to, the Wnt/β-catenin (the gene name *WNT* is a portmanteau of “Wingless” and “Int-1”) [42,43], fibroblast growth factor (FGF) signalling [43,44], Sonic Hedgehog (Shh) [42], TGF-β/BMP signalling [42,45], Notch signalling [42,46], Hippo, PI3K-Akt, Rap1, NF-κB, and cAMP pathways [47], androgen/cortisol–Wnt crosstalk, and transcription factors as well as alternative splicing regulators [48]. These are not the focus of this review; the emphasis is instead on the keratin and KAP genes that are expressed in the wool follicle.

The expression of keratin genes (*KRTs*) and keratin-associated protein genes (*KRTAPs*) produces the proteins that form the main structural components of the fibre. A detailed understanding of the expression patterns and phenotypic effects of these genes is critical to developing effective strategies to improve wool fibre qualities.

### 5.1. Wool Keratin Genes

The primary structural framework of wool fibres (as described above) is built from KIFs. These KIFs are formed by type I (acidic) and type II (neutral-basic) wool keratins [49], which are encoded by a subset of *KRT* genes that are active in the wool follicle. In sheep, at least 17 *KRTs* are reported to contribute to wool fibre structure, including 10 Type I (*KRT31*, *KRT32*, *KRT33A*, *KRT33B*, and *KRT34-KRT40*, excluding *KRT37*) and 7 Type II (*KRT81-KRT87*) genes, each with distinct expression profiles within the wool follicle [50,51,52,53]. The type II genes are clustered on chromosome 3, while the type I genes are located on chromosome 11, flanking the *KRTAP* gene cluster (two on one side and the remaining eight on the other).

The expression of wool *KRTs* is regulated both spatially and temporally. Some are expressed in the cuticle (e.g., *KRT40*, *KRT82*, and *KRT84*), or the cortex (e.g., *KRT31*, *KRT33A*, *KRT33B*, *KRT34*, *KRT36*, *KRT38*, *KRT39*, *KRT81*, *KRT83*, *KRT86*, and *KRT87*), while others are expressed transiently in both fibre structures (e.g., *KRT32*, *KRT35* and *KRT85*) [53]. The initial shared expression of selected *KRTs* likely supports early filament assembly and structural continuity and precedes divergence into the compartment-specific *KRT* expression that defines the final fibre architecture. In the cortex, there is co-expression of multiple type I and type II *KRTs* [53], with this facilitating the formation of a diversity of heterodimers. Given that the cortex constitutes the majority of the fibre volume, this molecular diversity likely plays a key role in shaping fibre structure and trait variability.

Despite the compartmentalised expression of the *KRTs* in the cuticle and cortex, to date no studies have reported differential expression of *KRT* genes between the ortho- and paracortex. This is in contrast to the *KRTAPs*, which exhibit region-specific expression. The apparent absence of differential *KRT* gene expression between the cortical regions suggests that their expression may be relatively stable and uniform across the cortical structures, with this likely reflecting functional constraints related to their essential role in maintaining fibre integrity. Although subtle variation in *KRT* gene expression may exist, it may be less pronounced than the greater variability and typically lower expression levels observed for *KRTAP* genes. However, further studies are needed to determine whether this reflects true uniformity, or whether it reflects current methodological limitations in measuring *KRT* expression in the various structures of the wool fibre.

To date, genetic variation has been examined in 10 of the 17 known wool keratin genes: six type I genes (*KRT31*, *KRT32*, *KRT33A*, *KRT34*, *KRT36*, and *KRT38*) [54,55,56,57,58] and four type II genes (*KRT81*, *KRT83*, *KRT84*, and *KRT85*) [57,59,60,61,62]. However, most of these investigations have only focused on parts of these genes, leaving portions of them unexplored. Consequently, the full extent of their nucleotide sequence variation is poorly understood.

All the wool keratin genes investigated to date exhibit genetic variation, primarily in the form of single nucleotide polymorphisms (SNPs). These SNPs occur in both the coding and non-coding regions. They include non-synonymous SNPs that change the amino acid sequence and potentially alter protein structure, and synonymous or non-coding SNPs that may influence gene expression or mRNA processing. All of these nucleotide sequence differences could contribute to variation in wool fibre traits.

Nucleotide sequence variation in several type I and type II keratin genes has been associated with a range of wool traits, supporting their functional relevance. For example, in Southdown × Merino-cross sheep, variation in *KRT31* has been linked to greasy fleece weight (GFW), clean fleece weight (CFW), and mean staple length (MSL) [55], while variation in *KRT81* has been associated with GFW and CFW [61].

### 5.2. Keratin-Associated Protein Genes

The KAPs form the matrix that surrounds and cross-links the keratin filaments within the wool fibres. Unlike the larger, intron-containing *KRTs*, the *KRTAPs* are typically small, intronless and exhibit greater sequence diversity. In sheep, the *KRTAP* repertoire has expanded to contain at least 102 genes [36], more than the 89 reported in humans [63,64,65]. While ovine and human *KRTAPs* are typically clustered and similarly arranged, species-specific differences in clustering patterns exist. For example, humans possess two *KRTAP5* clusters on the same chromosome [63], whereas sheep appear to have one cluster. The *KRTAP10* and *KRTAP12* genes form a single cluster in humans [63], while in sheep they appear to be split into two separate clusters, albeit located on the same chromosome. The HGT-*KRTAP* cluster also differs between the two species, spanning approximately 590 kb in humans and around 890 kb in sheep (our unpublished data). This expansion in size reflects the presence of additional HGT-*KRTAP* genes in sheep [66,67,68,69].

The *KRTAP* genes are broadly categorised into three major groups (HS, UHS and HGT), based on the amino acid composition of the proteins they encode, and further divided into twenty-six families based on nucleotide sequence similarity. Of these, twelve families (KAP1-KAP3, KAP11, KAP13, KAP15-KAP16, KAP23-KAP24, KAP26-KAP28) belong to the HS group, six families (KAP4-KAP5, KAP9-KAP10, KAP12, KAP17) belong to the UHS group, and eight families (KAP6-KAP8, KAP19-KAP22, KAP36) belong to the HGT group. The number of gene members within each family varies widely, with some having one single gene, while others, such as KAP5 and KAP10, have up to 12 members.

The *KRTAPs* exhibit spatial and temporal expression patterns during wool fibre development, both complementing and diverging from the expression patterns of the *KRTs*. However, our current knowledge is based on the limited subset of *KRTAPs* identified prior to the 1990s and involves only some members of the KAP1–KAP8 families. Among these, HGT-*KRTAPs* (e.g., KAP6–KAP8 families) are expressed early in fibre development, shortly after *KRT* expression begins. They are primarily restricted to the orthocortical region. Subsequently, HS-*KRTAPs* (e.g., KAP1–KAP3 families) appear in the cortical cells in the region complementary to that expressing KAP6-KAP8, but their expression soon extends across the entire cortex [37]. In contrast, UHS-*KRTAPs* (e.g., KAP4-KAP5 families) are expressed slightly later, with KAP4 expression confined to the paracortical cells [70], and KAP5 expression occurring late in cuticle cell differentiation [58,59,71,72]. These compartment-specific expression patterns likely contribute to the asymmetric architecture of wool fibres, influencing traits such as curvature and crimp.

Due to their small size and intronless nature, the *KRTAPs* are amenable to analysis of their entire coding sequence. All of the genes investigated to date exhibit polymorphism, although the extent and nature of that polymorphism vary between genes [73]. It must also be remembered that, of the billion plus sheep globally, the *KRTAPs* have only been studies in a few individuals and breeds. The gene polymorphisms in the *KRTAPs* include SNPs and insertion/deletions (indels), with allele numbers ranging from 2 to 14 per gene [73]. Most indels are located within the coding regions, and usually within or adjacent to tandem repeat elements. They typically preserve the reading frame, but an exception is observed for *KRTAP28-1*, where the indel variation involves a variable number of dinucleotide repeats, resulting in a frameshift near the C-terminus of the encoded protein [74].

While at times considered benign, the SNPs in the flanking regions and synonymous SNPs may affect gene expression or mRNA processing, while non-synonymous SNPs can lead to changes in amino acid sequences. Indels, when present, can cause more substantial structural alterations to the encoded proteins. Collectively, these variations can influence the cross-linking potential and matrix composition, thereby potentially affecting wool fibre characteristics.

Variation in many *KRTAPs* has been associated with a variety of wool fibre traits, reinforcing their functional importance. For example, in Southdown × Merino-cross sheep, variation in *KRTAP6-1* and *KRTAP6-3* have been associated with variation in mean fibre diameter (MFD) related traits [75,76]. Variation in *KRTAP8-1* has been associated with variations in mean fibre curvature (MFC) and mean staple strength (MSS) [77], and variation in *KRTAP15-1* has been associated with wool yield and fibre diameter standard deviation (FDSD) [78].

Early studies have provided valuable insights into the expression patterns and sequence variation in some *KRTAPs*, but many ovine *KRTAPs* have yet to be characterised, and research in this area has been limited. Renewed effort to investigate *KRTAPs*, including their identification, expression profiles in the wool follicle, genetic variation, and functional roles, are essential to advancing our understanding of the genetic basis of wool fibre variation. This effort must include the study of many more sheep, and of a wider range of breeds and wool types.

### 5.3. Integrated Effects of Keratin and KAP Genes

The structural components of wool fibre, keratins and KAPs, are encoded by numerous genes. Their genetic diversity underpins the polygenic nature of wool traits, where fibre characteristics likely result from the combined effects of many genetic loci. In this respect, it is notable that all the wool *KRTs* and *KRTAPs* investigated to date in sheep are polymorphic, and they often possess multiple alleles [36]. The aggregated variation across both the *KRTs* and *KRTAPs* therefore likely contributes to the wide phenotypic diversity observed in fibre traits. That is, most wool traits appear to be influenced by multiple genes, each with small to moderate effects. To better understand and improve fibre traits, a comprehensive evaluation of the full catalogue of *KRTs* and *KRTAPs* is therefore essential, particularly to identify those genes with larger effects.

Interestingly, *KRT* and *KRTAP* genes located in close proximity to each other often show different trait associations. This suggests limited linkage disequilibrium and functional divergence. For example, among the type I wool *KRTs*, *KRT31* and *KRT34* are found in close proximity on chromosome 1, but variation in them shows different associations with wool traits in the same flock: *KRT31* variation is linked to GFW, CFW, and MSL [55], whereas *KRT34* variation is associated with MFD, FDSD, and MSL [54]. A similar pattern is seen among nearby type II wool *KRTs*: variation in *KRT83* is associated with MFD, FDSD, coefficient of variation in fibre diameter (CVFD), MFC, prickle factor (PF), and yield [59], while *KRT85* is linked to GFW, CFW, and PF in the same population [60].

The same phenomenon appears to occur with the *KRTAPs*. For example, *KRTAP21-1* is associated with wool yield [79], whereas the adjacent *KRTAP21-2* is linked to MSL [80]. Within the KAP13 family, *KRTAP13-2* is associated with MFD and CVFD in heterotypic hair fibres from Chinese Tan sheep, but variation in *KRTAP13-4* has no such association [81].

Taken together, these observations suggest that genes that are physically close on the chromosome may nevertheless have distinct functions. It also suggests that the multiplicity of *KRTs* and *KRTAPs* in sheep may not reflect functional redundancy, but instead they have evolved specialised or divergent roles in fibre development and structure. Accordingly, both the individual and combined effects of these genes must be considered in fibre trait studies.

Gene-by-trait associations can also vary across sheep breeds or populations, highlighting the influence of genetic background. For example, *KRT33A* is linked to fleece weight, yield, MSL, MFC, crimp frequency, and core bulk in Perendale sheep [58]; to FDSD and MSS in Merino and Merino-cross sheep [82]; and to CFW, MFD, MSL, and MSS in several Egyptian sheep breeds [83]. Similarly, *KRTAP20-1* is associated with GFW, wool yield, MFD, FDSD, and PF in Southdown × Merino-cross sheep [84], but with MFC in the fine wool of Chinese Tan sheep [85]. These findings highlight the importance of considering breed- or population-specific genetic contexts, particularly variation in other *KRTs* and *KRTAPs*, in association studies.

The physical clustering of *KRT* and *KRTAP* genes can lead to linkage effects, where neighbouring genes may be co-inherited due to linkage disequilibrium. However, findings in sheep suggest that linkage disequilibrium within some clusters is weak, as neighbouring loci often exhibit different associations with wool traits (see discussion above). This pattern, which is somewhat unexpected given their physical proximity, might be explained by recombination or gene conversion events that disrupt linkage disequilibrium. While direct evidence of this remains limited, sequence analyses have suggested that such mechanisms may have occurred with the KAP1 gene family [86,87].

Tighter linkage may however exist in some clusters. Evidence from Longdong cashmere goats suggests that several physically adjacent genes are associated with the same traits. For example, three adjacent genes, *KRTAP22-2*, *KRTAP6-5,* and *KRTAP6-2*, have all been associated with MFD for goat fibres [88,89,90]. Similarly, *KRTAP28-1* and *KRTAP24-1* (also physically adjacent) have both been linked to goat fibre MFD [91,92]. These findings suggest that some gene clusters may exhibit coordinated associations with fibre traits, possibly reflecting localised linkage disequilibrium. Overall, more extensive investigation across gene clusters is needed to better understand these patterns and their implications for fibre traits.

From a breeding perspective, the polygenic and potentially linked architecture of wool traits presents both challenges and opportunities. The small effects of individual genes limit the impact of single-gene selection, making multi-loci approaches more appropriate for capturing the underlying genetic variation. Weak linkage among some clustered genes offers flexibility, allowing improvement of specific traits without unintended effects on others. Conversely, tight linkage among loci can facilitate selection when favourable alleles co-occur on the same extended haplotype but may hinder progress if favourable alleles are linked with unfavourable ones. In such cases, selection strategies must evaluate and manage these trade-offs.

Overall, the genetic architecture underlying wool traits reflects a complex interplay of multiple genes, physical clustering, recombination, and gene-specific effects. Broader and deeper genomic analyses across entire gene clusters and diverse breeds will be essential for understanding this complexity and enabling more precise and effective genetic improvement strategies.

## 6. Genetic Strategies for Improving Wool Quality

Wool fibres display variation in traits that affect fibre uniformity and consequently product quality. Fortunately, many important wool traits have moderate to high heritability (0.3 to 0.6+) [93,94], meaning that a substantial proportion of phenotypic variation is genetically determined and can be improved through genetic approaches, including selective breeding.

To date, this selection has progressed from simply selecting rams with desirable fibre traits for breeding, in the hope that these will be passed on to progeny, to a more precise approach using estimated breeding values (eBVs) and selection indices based upon selected and weighted eBVs for specific traits. In this respect, the most widely used scheme for wool is the Australian MERINOSELECT system [95]. This system uses what are referred to as ABVs (animal breeding values; but eBVs by definition), which are statistically derived estimates of an animal’s genetic merit for specific traits, such as MFD and CFW. These ABVs are combined into selection indices such as the Fine Wool (FW) or Wool Production (WP) indexes. The system allows breeding sires (and ewes) to be rated for their wool production potential, be it for use on any given Merino sheep stud for breeding, or when selling sheep genetics.

A 2024 summary of MERINOSELECT [96] describes the genetic gains made since 2000 across three classes of wool sheep breeders (low-micron, mid-micron, and high-micron), noting that the low-micron breeders have reduced MFD and improved gastrointestinal parasite resistance; the mid-micron breeders have had ‘balanced improvements across multiple traits including fat, muscle, fleece weight, fibre diameter, reproduction and wrinkle’; and the high-micron breeders have ‘achieved significant gains in fat and muscle but at the expense of fleece weight, micron, wool colour, and shear force’.

In contrast to eBV-based selection, two main DNA-based strategies have been pursued to improve wool traits: genetic modification and marker-assisted selection. While genetic modification depends on understanding the molecular genetics and regulatory mechanisms underlying wool fibre traits, marker-assisted selection relies on identifying robust genetic markers linked to desirable phenotypes, even if the underlying mechanisms are not fully understood.

### 6.1. Genetic Modification: High Potential, Limited Progress

Genetic modification (also known as genetic engineering) involves the manipulation of an organism’s DNA to alter specific traits. In the context of wool production, this approach holds promise for improving fibre quality by targeting genes that influence the structural and mechanical properties of the wool fibre. Unlike marker-assisted selection, which works within the limits of naturally occurring genetic variation, the approach allows for the direct modification of individual genes and enables the introduction of novel genetic changes that may not be achievable through natural selection. The most relevant targets include genes encoding wool fibre structural proteins, as well as other proteins involved in fibre development, protein cross-linking, and biochemical modifications that affect fibre properties.

#### 6.1.1. Early Transgenic Experiments

During the 1990s and early 2000s, a series of pioneering transgenic experiments in mice and sheep explored the potential to improve fibre quality. Initial studies in transgenic mice investigated the effects of altering the expression of hair protein genes. One early experiment introduced the type II wool keratin gene *K2.10* (now *KRT83*) under control of its native promoter [97]. Transgenic mice expressing various levels of this gene displayed a range of hair phenotypes, from normal to distorted and fragile hairs, with this depending on the transgene copy number [97,98]. High levels of expression caused structural defects and hair loss, suggesting the dosage sensitivity of hair protein networks and the need for balanced expression to maintain fibre integrity.

Subsequent studies examined the effect of exchanging promoters between wool protein genes. When a *KRTAP2* gene, normally expressed late in fibre formation, was driven by the *KRT83* (formerly *K2.10*) promoter, its protein was expressed earlier than usual in the cortex. Several mouse lines exhibited variable hair phenotypes, ranging from normal to unusual, with two lines showing hair loss and one displaying a striking stubble-like coat after the first hair cycle [37,99]. Ultrastructural analyses revealed abnormal accumulation of the KAP2 protein [99], reinforcing the important roles of both protein abundance and temporal regulation of expression during fibre development.

Further experiments used the *KRT83* promoter to drive expression of a non-hair protein, SV40 T-antigen, in the mouse hair cortex. This caused severe hair deformation and fragility [100], likely due to interference with endogenous transcription factors such as AP-2, as many hair *KRT* and *KRTAP* gene promoters contain putative AP-2 binding sites [37]. Together the above findings highlight the importance of precise and appropriate transcriptional regulation of fibre structural genes to ensure normal hair development and integrity.

Transgenic sheep overexpressing *KRTs* and *KRTAPs* have been generated to study the impact of altered gene expression on wool fibre characteristics. Although these transgenes were successfully expressed in wool follicles, the resulting phenotypes were generally unfavourable. For example, overexpressing *KRT83* (formerly *K2.10*) resulted in altered fleece traits such as reduced crimp, increased lustre, and elevated lanolin content, while overexpression of *KRTAP4-2*, *KRTAP5-1*, or *KRTAP6-1* (previously *KAP4.2*, *KAP5.1*, or *KAP6.1*, respectively) reduced fibre strength [101,102,103]. These results suggest that even when transgenes are targeted to wool follicles, ectopic or excessive expression can disrupt the delicate balance of structural protein interactions required for normal fibre development. They also highlight the importance of finely regulated gene expression in maintaining wool quality.

Collectively, these early genetic modification experiments demonstrated that even modest disruptions in the expression level or timing of wool structural gene expression can compromise fibre quality. They suggest that the keratins and KAPs function within an interdependent and dosage-sensitive network, and any imbalance in that network could lead to weakened fibres, abnormal morphology, or other defects.

Other attempts to enhance wool production via genetic modification have focused on the overexpression of growth-regulatory genes, such as the ovine insulin-like growth factor 1 and the growth hormone genes. While some transgenic lines showed modest increases in wool production, the results were often inconsistent and sometimes accompanied by adverse health effects [104,105,106]. In this respect, while studies in mice involving manipulation of key signalling pathways, such as the Wnt/β-catenin, Shh, Bone Morphogenetic Protein, and EDA/EDAR pathways, have provided valuable insights into follicle development and density regulation [107,108,109,110], applying these findings to improving wool in sheep remains challenging. Although the core follicular regulatory pathways appear to be conserved across species, the biological differences between mice and sheep limit the direct translation of findings from one species to the other.

Despite early proof-of-concept experiments, progress with genetic modification has been limited due to the molecular complexity of wool fibre development and our incomplete understanding of the genetic and regulatory mechanisms involved. This highlights the need to better define the pathways governing fibre trait development and variation, and to identify critical gaps in our knowledge.

#### 6.1.2. The Potential of CRISPR/Cas Editing for Wool Trait Improvement

Clustered Regularly Interspaced Short Palindromic Repeats (CRISPR) refers to a natural bacterial immune system that has been adapted into a powerful genome-editing technology. It functions together with a Cas nuclease (commonly Cas9), which is directed to a specific DNA target by a single-guide RNA (sgRNA). When the Cas9–sgRNA complex binds the target DNA, Cas9 introduces a double-stranded break. The cell repairs this break either by non-homologous end joining, which can introduce insertions or deletions, creating gene knockouts, or by homology-directed repair, which allows insertion or precise editing using a supplied DNA template. This makes CRISPR/Cas gene targeting specific, flexible, fast, and cost-effective.

The CRISPR/Cas editing approach has been used to engineer the loss of *FGF5* function, resulting in increased wool staple length in sheep [111]. While capable of precisely targeting genes, as with the more rudimentary transgenesis experiments described above, care is needed to ensure that the genes targeted are not pleiotropic, and that the engineering does not raise ethical or welfare concerns. In most jurisdictions, genetic engineering is regulated, and consumer acceptance of these practices is often an issue.

#### 6.1.3. Molecular Complexity and Knowledge Gaps in Wool Genetic Modification

Although the early transgenic experiments provided valuable insights into the roles of structural and regulatory genes, they also revealed the intricate nature of the wool fibre system. Wool fibre development is governed by the coordinated action of numerous genes, including many wool *KRTs* and a diverse catalogue of *KRTAPs*. These genes are expressed in tightly regulated spatial and temporal patterns during follicle development [53], with this indicating that fibre formation relies on a finely tuned gene expression programme. It appears that the timing, location, and relative levels of expression for various genes must be coordinated to produce normal fibre structure and function.

For genetic modification to be used in future to modify wool traits, it may therefore be necessary to first identify which genes influence specific fibre characteristics and to understand the extent to which gene expression levels can be altered without disrupting the integrity of the fibre. Importantly, not only does the absolute expression level of any given protein appear to be crucial, but so too does the balance between different structural proteins. Disruptions in this balance, as seen in early transgenic experiments involving overexpression of *KRT83* or various *KRTAP* genes [101,102,103], have led to abnormal fibre morphology and compromised fibre integrity. These findings highlight the importance of maintaining coordinated expression.

What is more, achieving this level of precision will undoubtedly be challenging. Many *KRTs* and *KRTAPs* are clustered in the genome [73], raising the possibility of co-regulation of individual genes or the presence of shared regulatory elements. Attempts to manipulate individual genes may therefore affect adjacent genes, or broader regulatory networks, and complex genomic organisation may therefore present a barrier to targeted genetic modification.

Further complexity also likely arises from post-translational modifications. For example, differential phosphorylation of keratins and KAPs has been observed between crimped and straight wool fibre [112]. This suggests that some wool traits may be epigenetically regulated, something that may be difficult to control by genetic modification. What is more, wool traits are typically affected by a wide range of environmental factors, not least the effect of nutrition, disease, and photoperiod. Although these factors are beyond the scope of this review, they cannot be ignored in the quest to obtain more consistent wool fibre.

Taken together, these biological and technical challenges are compounded by our limited understanding of the molecular and regulatory landscape governing wool fibre development. Many *KRTAP* genes remain unidentified, and the roles of the *KRT* and *KRTAP* genes are still unclear. Available gene expression data are sparse, and mechanisms controlling gene expression, post-transcriptional processes, and the effects of environmental factors beyond the level of phenotype are largely unknown.

These conceptual challenges highlight the need for a much deeper understanding of the gene networks, expression patterns, regulatory mechanisms, and protein interactions involved in wool fibre development. Alongside these knowledge gaps, technical limitations in gene manipulation and expression control further complicate efforts to achieve successful genetic modification of wool traits. We conclude that more foundational research is required before genetic modification approaches can be effectively applied to wool trait improvement.

### 6.2. Genetic Selection: Practical Approaches, Ongoing Challenges

When compared to genetic modification, marker-assisted selection [113] offers a more practical and readily adoptable strategy for improving wool traits. It is a breeding method that uses DNA markers (such as SNPs, natural variation in genes, or microsatellite sequences) that are linked to, or within genes that control traits of interest. Instead of relying only on observable traits or phenotypes, breeders can test animals at the DNA level to determine whether they carry favourable alleles, which can then be selected for breeding.

Marker-assisted selection relies on capturing the benefit of naturally occurring genetic variation rather than direct manipulation of the genome. This makes it compatible with existing breeding programmes that utilise phenotypic selection and/or eBVs. Unlike genetic modification, it generally does not raise public and consumer ire. However, its success will depend on multiple factors, from the biological relevance of gene–trait associations to how genetic variation is analysed, and then how validated markers are integrated into breeding strategies.

Genomic BLUP (gBLUP) is a statistical method for genomic selection in breeding programmes that uses SNP marker data (omics data) to predict the genetic merit of individuals for complex traits [114,115]. In the simpler best linear unbiased predictor (BLUP) approach to genetic analysis, which is informed by phenotypic data, the method replaces the traditional pedigree-based relationship matrix with a genomic relationship matrix calculated from SNP genotypes. In this way, it provides more accurate eBVs by directly accounting for genetic relationships across the genome. gBLUP and SNP data from a 50 k SNP chip (approximately 50,000 genetic markers) are used by the MERINOSELECT system in Australia to evaluate Merino sheep. It produces what are known as Australian Sheep Breeding Values (ASBVs) using this evaluation approach, and enables more accurate selection of animals based on their genetic merit for various traits.

While gBLUP has shown value in livestock breeding, its application to wool traits remains challenging. Standard SNP chips may not adequately represent the genetic variation in *KRT* and *KRTAP* genes [36], and the inherently complex nature of wool traits further limits the accuracy of genomic prediction. Given these constraints, this section focuses on marker-assisted selection, which provides a practical, gene-informed approach to improving wool traits.

#### 6.2.1. Ensuring Biological Plausibility in Gene–Trait Associations

The success of marker-assisted selection depends on using genetic markers with robust and biologically plausible associations with fibre traits. When selecting genes for association studies or interpreting such associations, it is crucial to assess whether the gene involved has a plausible biological role related to the target trait.

For example, if a gene is claimed to influence egg quality based on an observed association, we would expect it to affect a component of the egg such as the yolk, albumen, or shell, which define egg quality. If no such functional effect is observed, the association may be questionable. Similarly, genes linked to wool fibre traits should plausibly affect wool proteins (e.g., the keratins or KAPs) or follicle development processes, whether through changes in structure, abundance, assembly, or regulation. Associations lacking such biological relevance should be treated with greater caution.

Two main strategies exist for identifying gene–trait associations: the candidate gene approach and the omics-based approach. The candidate gene approach targets genes with established or suspected biological relevance to fibre traits and remains the more reliable and practical strategy for gene–trait association studies. In contrast, the omics-based approach (e.g., using SNP chips) takes a genome- or system-wide perspective and relies on large-scale statistical correlations with traits of interest rather than on prior biological knowledge of particular genes. This approach has been applied in practice, for example, in Australia, where SNP chip data are used to select sheep with superior wool genetics for breeding. While omics approaches can lead to the identification of superior pedigrees, they usually rely on markers derived from genome-wide scans rather than on genes of known or well-understood function, and thus the markers often act as anonymous indicators of superior genetics.

Although omics studies have reported many genes to be associated with wool traits, numerous candidate genes inferred from these associations lack obvious biological relevance to fibre formation or development, or they do not specifically affect wool traits without having other pleiotropic effects that may be untoward. While this does not negate their importance, it does suggest that caution is warranted when interpreting omics results, and importantly that these findings require rigorous validation before they can be used in selective breeding. Omics approaches can be valuable for describing genetic diversity and identifying selection signatures, and thus they are useful for the management and sustainable utilisation of sheep genetic resources [116].

#### 6.2.2. Choosing the Appropriate Level of Genetic Variation

Genetic associations can be explored at distinct levels of genetic variation, ranging from SNPs to haplotypes spanning one or multiple genes. Wool *KRT* and *KRTAP* genes are often physically clustered and functionally related [73], making it appealing to analyse variation at the multi-gene haplotype level. These haplotypes may capture the combined effects of neighbouring loci, offering a more integrated genetic signal.

However, several practical limitations hinder the routine use of multi-gene haplotypes. Most of the *KRT* and *KRTAP* genes are multi-allelic [73], and as discussed earlier, even closely located genes can exhibit distinct associations with different fibre traits. This suggests that despite physical proximity, these loci may not be in strong linkage disequilibrium, leading to a large number of possible multi-gene haplotypes. Consequently, detecting associations at this level of genetic complexity will require large sample sizes to achieve adequate statistical power. Moreover, accurately detecting and phasing these complex haplotypes remains technically challenging. Existing genotyping platforms are not well-suited for this level of resolution, and while long-read sequencing holds promise, it is not yet cost-effective or practical for large-scale routine use as might be required to understand high levels of trait variation.

Given these constraints, the most feasible and biologically sound strategy is to focus on within-gene haplotypes (also referred to as alleles or variants). These haplotypes represent combinations of SNPs across the entire gene or a variable portion of the gene (if the gene is too large for full analysis) and can capture functionally relevant variation. Since each wool protein gene encodes a specific structural component of the wool fibre, analysing variation at this level is justifiable and technically achievable. Techniques such as PCR-SSCP offer reliable and cost-effective means of genotyping these haplotypes [117].

In contrast, SNP-level association analysis has limited utility for wool trait association for several reasons. First, most wool protein genes are multi-allelic [73]. Simplifying this diversity into biallelic SNPs can mask meaningful associations. Second, the high sequence similarity among related genes makes it difficult to assign SNPs to specific loci, which can compromise genotyping accuracy [36]. Third, it is often the overall structure of the protein, rather than individual amino acid residues, that determines how the protein interacts and arranges with others, thereby affecting wool fibre structure [36]. As a result, focusing on individual SNPs may fail to capture the functional complexity underlying wool fibre formation.

#### 6.2.3. Integrating Genetic Markers into Breeding Programmes

The ultimate goal of gene–trait association research is to translate genetic insights into practical tools for improving wool quality. For marker-assisted selection to be effective, genetic markers must be robustly validated, economically viable, and effectively integrated into breeding strategies. This has been achieved with genomic selection approaches being used to improve wool traits in Australia [95], and for traits like growth, carcass and eating quality, reproduction, and health [118]. Individual genes are being used to augment sheep breeding, with DNA typing services provided by testing laboratories like the Lincoln University Gene-Marker Laboratory [119].

Prior to integration, candidate markers should undergo thorough validation across diverse breeds, populations, and production environments to ensure their reliability and consistency. This includes testing for reproducible associations with target traits and evaluating their stability under varying environmental conditions. Once validated, markers are prioritised based on several key criteria, including effect size, strength of association, linkage disequilibrium with other markers (to avoid redundancy and optimise marker panels), the technical feasibility of genotyping, and the overall cost-effectiveness.

Selected markers can then be incorporated into breeding programmes by including them in selection indices or by using them to enhance the accuracy of estimated breeding values, thereby improving the precision of selection. While integrating validated gene markers has the potential to improve selection accuracy and accelerate genetic gain, the complex genetic architecture of wool traits means that ongoing research is necessary to refine marker selection and develop effective approaches.

## 7. Trade-Offs and Synergies in Wool Trait Improvement

As wool marker-assisted selection programmes evolve, a key challenge is managing potential trade-offs, whether between different wool traits themselves or between wool traits and other economically important sheep production traits such as growth, meat production, and reproduction. This issue is particularly relevant in modern sheep production systems, where meat has overtaken wool as the primary value proposition, and wool is considered to be a secondary product of sheep farming [120]. Addressing these genetic conflicts is essential to achieving balanced and sustainable genetic progress.

### 7.1. Trade-Offs and Synergies Among Wool Traits

Of the wool traits, CFW and MFD are two of the most economically important wool traits. A common concern is whether selection to improve one may negatively affect the other. Specifically, increased CFW has sometimes been associated with increased MFD, which can reduce the value of wool [121,122,123]. This trade-off is partly explained by fibre physics: for a fixed number and length of fibres, finer fibres would be expected to contribute less to total fleece weight than coarser ones.

However, this relationship is not inevitable. Evidence from well-designed breeding programmes, such as the Trangie QPLU$ project, shows that simultaneous improvement of CFW and MFD is possible through selection without detriment to either trait [124]. Long-term studies, such as Ramos et al. [125], further confirm that balanced selection can reduce fibre diameter and maintain or increase fleece weight over time.

The genetic correlations underlying wool traits depend largely on pleiotropy and genetic linkage. If a gene influences multiple traits, its effects may all be favourable (creating positive synergies) or partly opposing (creating trade-offs). Similarly, different genes that are linked on the chromosome can also cause correlated responses, even if their functions are unrelated. In contrast, traits controlled by independent loci can be improved simultaneously without adverse interactions. As an example, a genetic association study revealed that specific *KRTAP1-2* variants are associated with both increased fleece weight and finer fibre diameter [126], results that are consistent with the above findings of Mortimer et al. [124] and Ramos et al. [125], and with this suggesting that favourable genetic variants can enhance both wool quantity and quality.

The underlying genetic architecture of wool traits is complex and polygenic, with numerous small-effect genes often clustered together. This complexity limits the effectiveness of traditional phenotypic selection, which may not easily separate correlated effects between traits. Marker-assisted selection offers a promising path forward by enabling breeders to identify and select for beneficial variants, while avoiding those with negative impacts. This targeted approach can enhance selection efficiency and facilitate more balanced genetic improvement across multiple wool traits of value.

### 7.2. Potential Effects of Wool Trait Selection on Production and Reproductive Traits

A common concern is whether selection for wool traits, particularly CFW and MFD, might adversely impact growth, carcass traits, or reproductive performance. This question is particularly relevant in both dual-purpose and wool-focused production systems, where genetic improvement must strike a balance between fleece value, meat production, and reproductive efficiency to ensure overall sheep farming profitability.

Genetically and physiologically, wool, growth, and reproductive traits are largely controlled by different genes and pathways, suggesting that they can be improved concurrently without inherent antagonism. However, all traits rely on shared metabolic resources, especially energy and nutrients. Under suboptimal nutritional or environmental conditions, competition for these limited resources may lead to physiological trade-offs, where improvement in one trait could come at the expense of another. Therefore, effective selection programmes must consider not only the genetic makeup but also the importance of providing appropriate environmental and nutritional support to fully express genetic potential across all traits.

Empirical evidence supports the feasibility of simultaneous genetic improvement of wool, growth, carcass, and reproductive traits. Mortimer et al. [127] reported generally favourable genetic correlations between wool and carcass traits in Merino sheep. While selection for increased CFW was associated with decreased carcass fat and dressing percentage, it had negligible impact on carcass muscle levels. Similarly, selection for lower MFD confirmed some unfavourable correlations with carcass weight and muscle mass, but these were minor. Mortimer et al. [128] also established moderate and favourable genetic correlations (~0.5) between fleece weight and live weight, and weak correlations between MFD and ultrasound traits such as eye muscle depth and fat depth. In Columbia and Targhee sheep, Hanford et al. [129,130] reported low to moderate positive genetic correlations between wool and growth traits, and low negative correlations between fleece weight and reproductive traits, suggesting minimal antagonism. Mortimer et al. [131] revealed low to negligible genetic correlations between wool traits and a range of meat quality and nutritional traits, indicating that selection for wool traits is unlikely to compromise meat quality.

Long-term selection studies provide further support. Ramos et al. [125] reported sustained genetic gains in both fleece weight and MFD over nearly two decades in a Merino population, with no adverse effects on ewe body condition or reproductive performance. The modest gains in reproduction were attributed to their exclusion from the selection index, rather than to unfavourable genetic correlations. This highlights the importance of including reproductive traits in selection objectives, rather than assuming inherent biological trade-offs.

A practical outcome from a dual-purpose breeding programme demonstrates the potential for co-improvement. An industry trial has identified lambs with both high-value wool traits and desirable eating quality, suggesting the concurrent genetic improvement of eating quality and wool quality is possible [132]. While based on a single trial, the results support the principle that, with appropriate selection strategies and proper management, simultaneous genetic progress across multiple economically important traits can be achieved.

Current evidence from quantitative genetic studies, long-term selection experiments, and practical breeding programmes reveals that selection for improved wool traits does not inherently compromise growth or reproductive performance. When breeding programmes are carefully designed to consider genetic correlations and are supported by appropriate management, it is both feasible and beneficial to pursue simultaneous improvement in wool, meat, and reproductive traits, thereby enhancing the overall productivity, resilience, and profitability of sheep flocks across production systems.

## 8. Conclusions and Future Directions

Wool is a unique fibre, valued for its exceptional functional properties, as well as its health and environmental benefits, which make it suitable as a sustainable fibre for many uses. These qualities arise from its sophisticated ordered structure, which is shaped by intricate molecular mechanisms that remain poorly understood and involve numerous genes, only a fraction of which have been identified.

Recent progress in wool genetics has improved our understanding of fibre formation and trait variation, and it opens opportunities for targeted genetic improvement, whether through genomic selection or marker-assisted selection using variation in keratin and keratin-associated protein genes. This lays a solid foundation for the improvement of wool. While genetic modification, especially precise gene-editing approaches like CRISPR/Cas, offer future potential, much greater focus is needed on identifying and characterising the genes involved in fibre formation and development. This should include research into the molecular mechanisms underlying wool trait variation. These efforts will help ensure the long-term viability, adaptability, and competitiveness of wool production in the face of evolving economic and environmental contexts.

## References

[1] Kuffner H., Popescu C., Kozłowski R.M., Mackiewicz-Talarczyk M. (2012). Wool fibres. Handbook of Natural Fibres.

[2] Wool Notes, Issue 3, A Summary of Wool Textile Information, Including Notes and Interesting Wool Facts. 2024. International Wool Textile Organisation 2024. https://iwto.org/sheep-wool/about-sheep/.

[3] Datta M., Basu G., Das S., Sreekala M.S., Ravindran L., Goda K., Thomas S. (2023). Wool, a natural biopolymer: Extraction and structure–property relationships. Handbook of Natural Polymers.

[4] Latif E. A review of low energy thermal insulation materials for building applications. Proceedings of the 2nd International Conference on Green Energy and Environmental Technology (ICGEET).

[5] Berardi U., Iannace G. (2015). Acoustic characterization of natural fibers for sound absorption applications. Build. Environ..

[6] Ormondroyd G., Curling S., Mansour E., Hill C. (2017). The water vapour sorption characteristics and kinetics of different wool types. J. Text. Inst..

[7] Why Wool Feels warm. My Textile Notes. https://mytextilenotes.blogspot.com/2009/10/why-wool-feels-warm.html.

[8] Mansour E. (2018). Wool Fibres for the Sorption of Volatile Organic Compounds (VOCs) from Indoor Air. Ph.D. Thesis.

[9] McNeil S. The Removal of Indoor air Contaminants by Wool Textiles. Technical Bulletin, 2015. https://www.researchgate.net/profile/Steve-Mcneil/publication/353757473_The_Removal_of_Indoor_Air_Contaminants_by_Wool_Textiles/links/610f02bf0c2bfa282a2f469c/The-Removal-of-Indoor-Air-Contaminants-by-Wool-Textiles.pdf.

[10] Johnson N., Wood E., Ingham P., McNeil S., McFarlane I. (2003). Wool as a technical fibre. J. Text. Inst..

[11] Sid Masri M., Reuter F.W., Friedman M. (1974). Interaction of wool with metal cations. Text. Res. J..

[12] Laurie S.H., Barraclough A. (1979). Use of waste wool for the removal of mercury from industrial effluents, particularly those from the chlor-alkali industry. Int. J. Environ. Stud..

[13] Allahkarami E., Allahkarami E., Rezai B. (2025). A brief review on utilizing natural adsorbents for microplastic removal from wastewater: A sustainable approach to environmental protection. Results Eng..

[14] International Wool Textile Organisation Wool & Fire. https://iwto.org/wp-content/uploads/2020/04/IWTO_Wool-Fire.pdf.

[15] London Fire Brigade Firefighters’ uniforms. London Fire Brigade Museum. https://www.london-fire.gov.uk/museum/london-fire-brigade-history-and-stories/equipment-and-uniforms/firefighters-uniforms/.

[16] Gambichler T., Rotterdam S., Altmeyer P., Hoffmann K. (2001). Protection against ultraviolet radiation by commercial summer clothing: Need for standardised testing and labelling. BMC Dermatol..

[17] CHARACTERISTICS OF WOOL Fact Sheet. https://www.wool.ca/images/uploads/files/care/wool-fact-sheets-charcteristics.pdf.

[18] Su J., Dailey R., Zallmann M., Leins E., Taresch L., Donath S., Heah S., Lowe A. (2017). Determining effects of superfine sheep wool in INfantile eczema (DESSINE): A randomized paediatric crossover study. Brit. J. Dermatol..

[19] Spelman L., Supranowicz M.J., Davidson K.A., Johnston J.J., Yau B., Holland T.L. (2018). An investigator blinded, clinical trial assessing the efficacy of superfine merino wool base layer garments (SMWBG) in children with atopic dermatitis (AD) measuring SCORAD1, EASI2, POEM3 and DSA4 scores. Biomed. J. Sci. Tech. Res..

[20] Fowler J.F., Fowler L.M., Lorenz D. (2019). Effects of merino wool on atopic dermatitis using clinical, quality of life, and physiological outcome measures. Dermatitis.

[21] Zallmann M., Smith P.K., Tang M.L., Spelman L.J., Cahill J.L., Wortmann G., Katelaris C.H., Allen K.J., Su J.C. (2017). Debunking the myth of wool allergy: Reviewing the evidence for immune and non-immune cutaneous reactions. Acta Derm. Venereol..

[22] Shin M., Halaki M., Swan P., Ireland A.H., Chow C.M. (2016). The effects of fabric for sleepwear and bedding on sleep at ambient temperatures of 17 °C and 22 °C. Nat. Sci. Sleep.

[23] Chow C.M., Shin M., Mahar T.J., Halaki M., Ireland A. (2019). The impact of sleepwear fiber type on sleep quality under warm ambient conditions. Nat. Sci. Sleep.

[24] Li X., Halaki M., Chow C.M. (2024). How do sleepwear and bedding fibre types affect sleep quality: A systematic review. J. Sleep Res..

[25] The Woolmark Company. (n.d.) Where Does Carbon Come From?. https://www.woolmark.com/globalassets/_06-new-woolmark/_industry/research/factsheets/gd2405-where-does-carbon-come-from_122.pdf.

[26] Wiedemann S.G., Biggs L., Nebel B., Bauch K., Laitala K., Klepp I.G., Swan P.G., Watson K. (2020). Environmental impacts associated with the production, use, and end-of-life of a woollen garment. Int. J. Life Cycle Assess..

[27] Allen M.R., Shine K.P., Fuglestvedt J.S., Millar R.J., Cain M., Frame D.J., Macey A.H. (2018). A solution to the misrepresentations of CO2-equivalent emissions of short-lived climate pollutants under ambitious mitigation. npj Clim. Atmos. Sci..

[28] Hodgson A., Leighs S.J., van Koten C. (2023). Compostability of wool textiles by soil burial. Text. Res. J..

[29] Collie S., Brorens P., Hassan M.M., Fowler I. (2024). Marine biodegradation behavior of wool and other textile fibers. Water Air Soil Poll..

[30] Laitala K., Klepp I.G. (2016). Wool wash: Technical performance and consumer habits. Tenside Surfact. Det..

[31] Caven B., Redl B., Bechtold T. (2018). An investigation into the possible antibacterial properties of wool fibers. Text. Res. J..

[32] Rippon J.A., Lewis D.M., Rippon J.A. (2013). The structure of wool. The Coloration of Wool and Other Keratin Fibres.

[33] Connell D.L., Heywood D. (2003). Chapter 11. Wool finishes: The control of shrinkage. Textile Finishing.

[34] Mercer E. (1954). The relation between external shape and internal structure of wool fibers. Text. Res. J..

[35] Harland D.P., Vernon J.A., Woods J.L., Nagase S., Itou T., Koike K., Scobie D.A., Grosvenor A.J., Dyer J.M., Clerens S. (2018). Intrinsic curvature in wool fibres is determined by the relative length of orthocortical and paracortical cells. J. Exp. Biol..

[36] Zhou H., Bai L., Li S., Li W., Wang J., Tao J., Hickford J.G. (2024). Genetics of wool and cashmere fibre: Progress, challenges, and future research. Animals.

[37] Powell B.C., Rogers G.E. (1997). The role of keratin proteins and their genes in the growth, structure and properties of hair. EXS.

[38] Plowman J.E., Paton L.N., Bryson W.G. (2007). The differential expression of proteins in the cortical cells of wool and hair fibres. Exp. Dermatol..

[39] Rippon J.A., Evans D.J., Kozłowski R.M., Mackiewicz-Talarczyk M. (2020). Improving the properties of natural fibres by chemical treatments. Handbook of Natural Fibres.

[40] Popescu C., Höcker H. (2007). Hair—The most sophisticated biological composite material. Chem. Soc. Rev..

[41] Leeder J.D. (1984). Wool: Nature’s Wonder Fibre.

[42] Rishikaysh P., Dev K., Diaz D., Qureshi W.M., Filip S., Mokry J. (2014). Signaling involved in hair follicle morphogenesis and development. Int. J. Mol. Sci..

[43] Zhang R., Li Y., Jia K., Xu X., Li Y., Zhao Y., Zhang X., Zhang J., Liu G., Deng S. (2020). Crosstalk between androgen and Wnt/β-catenin leads to changes of wool density in FGF5-knockout sheep. Cell Death Dis..

[44] Xu X.L., Wu S.J., Qi S.Y., Chen M.M., Liu Z.M., Zhang R., Zhao Y., Liu S.Q., Zhou W.D., Zhang J.L. (2024). Increasing GSH-Px activity and activating Wnt pathway promote fine wool growth in FGF5-edited sheep. Cells.

[45] Lv X., Chen L., He S., Liu C., Han B., Liu Z., Yusupu M., Blair H., Kenyon P., Morris S. (2020). Effect of nutritional restriction on the hair follicles development and skin transcriptome of Chinese Merino sheep. Animals.

[46] Xavier S.P., Gordon-Thomson C., Wynn P.C., McCullagh P., Thomson P.C., Tomkins L., Mason R.S., Moore G.P. (2013). Evidence that Notch and Delta expressions have a role in dermal condensate aggregation during wool follicle initiation. Exp. Dermatol..

[47] Li C., Feng C., Ma G., Fu S., Chen M., Zhang W., Li J. (2022). Time-course RNA-seq analysis reveals stage-specific and melatonin-triggered gene expression patterns during the hair follicle growth cycle in Capra hircus. BMC Genom..

[48] Yuan X., Meng K., Wang Y., Wang Y., Pan C., Sun H., Wang J., Li X. (2024). Unlocking the genetic secrets of Dorper sheep: Insights into wool shedding and hair follicle development. Front. Vet. Sci..

[49] Fraser R.B., Parry D.A. (2018). Structural hierarchy of trichocyte keratin intermediate filaments. Adv. Exp. Med. Biol..

[50] Powell B., Crocker L., Rogers G. (1992). Hair follicle differentiation: Expression, structure and evolutionary conservation of the hair type II keratin intermediate filament gene family. Development.

[51] Powell B.C., Crocker L.A., Rogers G.E. (1993). Complete sequence of a hair-like intermediate filament type II keratin gene. DNA Seq..

[52] Wilson B.W., Edwards K.J., Sleigh M.J., Byrne C.R., Ward K.A. (1988). Complete sequence of a type-I microfibrillar wool keratin gene. Gene.

[53] Yu Z., Wildermoth J.E., Wallace O.A., Gordon S.W., Maqbool N.J., Maclean P.H., Nixon A.J., Pearson A.J. (2011). Annotation of sheep keratin intermediate filament genes and their patterns of expression. Exp. Dermatol..

[54] Chai W., Zhou H., Gong H., Hickford J.G. (2022). Variation in the ovine *KRT34* promoter region affects wool traits. Small Rumin. Res..

[55] Chai W., Zhou H., Gong H., Wang J., Luo Y., Hickford J.G. (2019). Nucleotide variation in the ovine *KRT31* promoter region and its association with variation in wool traits in Merino-cross lambs. J. Agric. Sci..

[56] Chen Z., Zhao F., He Z., Sun H., Xi Q., Yu X., Ding Y., An Z., Wang J., Liu X. (2024). Expression localization of the *KRT32* gene and its association of genetic variation with wool traits. Curr. Issues Mol. Biol..

[57] Sulayman A., Tursun M., Sulaiman Y., Huang X., Tian K., Tian Y., Xu X., Fu X., Mamat A., Tulafu H. (2018). Association analysis of polymorphisms in six keratin genes with wool traits in sheep. Asian-Australas. J. Anim. Sci..

[58] Sumner R., Forrest R., Zhou H., Henderson H., Hickford J.G. (2013). Association of the *KRT33A* (formerly *KRT1.2*) gene with live-weight and wool characteristics in yearling Perendale sheep. Proc. N. Z. Soc. Anim. Prod..

[59] Chai W., Zhou H., Forrest R.H., Gong H., Hodge S., Hickford J.G. (2017). Polymorphism of *KRT83* and its association with selected wool traits in Merino-cross lambs. Small Rumin. Res..

[60] Chai W., Zhou H., Gong H., Wang C., Hickford J.G. (2024). Variation in the exon 3-4 region of ovine *KRT85* and its effect on wool traits. Animals.

[61] Li W., Bai L., Zhou H., Zhang Z., Ma Z., Wu G., Luo Y., Tanner J., Hickford J.G. (2024). Ovine *KRT81* variants and their influence on selected wool traits of commercial value. Genes.

[62] Yu X., Li S., Zhou H., Zhao F., Hu J., Wang J., Liu X., Li M., Zhao Z., Hao Z. (2024). Spatiotemporal expression and haplotypes identification of KRT84 gene and their association with wool traits in Gansu Alpine Fine-Wool sheep. Genes.

[63] Rogers M.A., Schweizer J. (2005). Human KAP genes, only the half of it? Extensive size polymorphisms in hair keratin-associated protein genes. J. Investig. Dermatol..

[64] Rogers M.A., Winter H., Langbein L., Wollschläger A., Praetzel-Wunder S., Jave-Suarez L.F., Schweizer J. (2007). Characterization of human KAP24.1, a cuticular hair keratin-associated protein with unusual amino-acid composition and repeat structure. J. Investig. Dermatol..

[65] Rogers M.A., Langbein L., Praetzel Wunder S., Giehl K. (2008). Characterization and expression analysis of the hair keratin associated protein KAP26.1. Brit. J. Dermatol..

[66] Gong H., Zhou H., Dyer J., Hickford J.G.H. (2014). The sheep KAP8-2 gene, a new KAP8 family member that is absent in humans. SpringerPlus.

[67] Gong H., Zhou H., Wang J., Li S., Luo Y., Hickford J.G. (2019). Characterisation of an ovine keratin associated protein (KAP) gene, which would produce a protein rich in glycine and tyrosine, but lacking in cysteine. Genes.

[68] Zhou H., Gong H., Wang J., Dyer J.M., Luo Y., Hickford J.G. (2016). Identification of four new gene members of the KAP6 gene family in sheep. Sci. Rep..

[69] Zhou H., Li W., Bai L., Wang J., Luo Y., Li S., Hickford J.G. (2023). Ovine *KRTAP36-2*: A new keratin-associated protein gene related to variation in wool yield. Genes.

[70] Fratini A., Powell B.C., Hynd P.I., Keough R.A., Rogers G.E. (1994). Dietary cysteine regulates the levels of messenger-rnas encoding a family of cysteine-rich proteins of wool. J. Investig. Dermatol..

[71] Jenkins B.J., Powell B.C. (1994). Differential expression of genes encoding a cysteine-rich keratin family in the hair cuticle. J. Investig. Dermatol..

[72] MacKinnon P., Powell B., Rogers G. (1990). Structure and expression of genes for a class of cysteine-rich proteins of the cuticle layers of differentiating wool and hair follicles. J. Cell Biol..

[73] Zhou H., Gong H., Wang J., Luo Y., Li S., Tao J., Hickford J.G. (2021). The complexity of the ovine and caprine keratin-associated protein genes. Int. J. Mol. Sci..

[74] Bai L., Wang J., Zhou H., Gong H., Tao J., Hickford J.G. (2019). Identification of ovine *KRTAP28-1* and its association with wool fibre diameter. Animals.

[75] Zhou H., Gong H., Li S., Luo Y., Hickford J.G.H. (2015). A 57-bp deletion in the ovine KAP6-1 gene affects wool fibre diameter. J. Anim. Breed. Genet..

[76] Li S., Zhou H., Gong H., Zhao F., Wang J., Luo Y., Hickford J.G. (2017). Variation in the ovine KAP6-3 gene (*KRTAP6-3*) is associated with variation in mean fibre diameter-associated wool traits. Genes.

[77] Gong H., Zhou H., Li W., Wang J., Li S., Luo Y., Hickford J.G.H. (2019). Variation in ovine *KRTAP8-1* is associated with variation in wool fibre staple strength and curvature. J. Agric. Sci..

[78] Li W., Gong H., Zhou H., Wang J., Liu X., Li S., Luo Y., Hickford J.G.H. (2018). Variation in the ovine keratin-associated protein 15-1 gene affects wool yield. J. Agric. Sci..

[79] Li S., Zhou H., Gong H., Zhao F., Wang J., Liu X., Hu J., Luo Y., Hickford J.G. (2019). Identification of the ovine keratin-associated protein 21-1 gene and its association with variation in wool traits. Animals.

[80] Li S., Zhou H., Gong H., Zhao F., Wang J., Liu X., Hu J., Luo Y., Hickford J.G. (2020). The mean staple length of wool fibre is associated with variation in the ovine keratin-associated protein 21-2 gene. Genes.

[81] Bai L., Zhou H., He J., Tao J., Hickford J.G. (2024). Characterisation of three ovine *KRTAP13* family genes and their association with wool traits in Chinese Tan sheep. Animals.

[82] Itenge T., Hickford J., Forrest R., McKenzie G., Frampton C. (2010). Association of variation in the ovine KAP1.1, KAP1.3 and K33 genes with wool traits. Int. J. Sheep Wool Sci..

[83] Farag I., Darwish H., Darwish A., Eshak M., Ahmed R. (2018). Genetic polymorphism of *KRT1.2* gene and its association with improving of some wool characteristics in Egyptian sheep. Asian J. Sci. Res..

[84] Gong H., Zhou H., Bai L., Li W., Li S., Wang J., Luo Y., Hickford J.G. (2019). Associations between variation in the ovine high glycine-tyrosine keratin-associated protein gene *KRTAP20-1* and wool traits. J. Anim. Sci..

[85] Bai L., Zhou H., Tao J., Hickford J.G. (2024). Effects of *KRTAP20-1* gene variation on wool traits in Chinese Tan sheep. Genes.

[86] Rogers G.R., Hickford J.G.H., Bickerstaffe R. (1994). Polymorphism in two genes for B2 high sulfur proteins of wool. Anim. Genet..

[87] Zhou H., Visnovska T., Gong H., Schmeier S., Hickford J., Ganley A.R. (2019). Contrasting patterns of coding and flanking region evolution in mammalian keratin associated protein-1 genes. Mol. Phylogenet. Evol..

[88] Chen Z., Cao J., Zhao F., He Z., Sun H., Wang J., Liu X., Li S. (2023). Identification of the keratin-associated protein 22-2 gene in the Capra hircus and association of its variation with cashmere traits. Animals.

[89] Li S., Xi Q., Zhao F., Wang J., He Z., Hu J., Liu X., Luo Y. (2021). A highly polymorphic caprine keratin-associated protein gene identified and its effect on cashmere traits. J. Anim. Sci..

[90] Cao J., Wang J., Zhou H., Hu J., Liu X., Li S., Luo Y., Hickford J.G. (2022). Sequence variation in caprine *kRTAP6-2* affects cashmere fiber diameter. Animals.

[91] Wang J., Zhou H., Hickford J.G., Zhao M., Gong H., Hao Z., Shen J., Hu J., Liu X., Li S. (2020). Identification of caprine *KRTAP28-1* and its effect on cashmere fiber diameter. Genes.

[92] Wang J., Zhou H., Luo Y., Zhao M., Gong H., Hao Z., Hu J., Hickford J.G. (2019). Variation in the caprine KAP24-1 gene affects cashmere fibre diameter. Animals.

[93] Mortimer S.I., Atkins K. (1989). Genetic evaluation of production traits between and within flocks of Merino sheep. I. Hogget fleece weights, body weight and wool quality. Crop Pasture Sci..

[94] Safari E., Fogarty N., Gilmour A. (2005). A review of genetic parameter estimates for wool, growth, meat and reproduction traits in sheep. Livest. Prod. Sci..

[95] Sheep Genetics. MERINOSELECT. https://www.sheepgenetics.org.au/resources/merinoselect/.

[96] (2024). Australian Wool Innovation. *MERINOSELECT ASBVs by Fibre Diameter and Their Genetic Trends*; Beyond the Bale. Issue 101. https://www.wool.com/globalassets/wool/beyond-the-bale/issue-101-december-2024/btb-2024_december_web.pdf.

[97] Powell B.C., Rogers G.E. (1990). Cyclic hair-loss and regrowth in transgenic mice overexpressing an intermediate filament gene. EMBO J..

[98] Powell B.C., Nesci A., Rogers G.E. (1991). Regulation of keratin gene expression in hair follicle differentiation. Ann. N. Y. Acad. Sci..

[99] Rogers G.E., Powell B.C. (1993). Organization and expression of hair follicle genes. J. Investig. Dermatol..

[100] Keough R., Powell B., Rogers G. (1995). Targeted expression of SV40 T antigen in the hair follicle of transgenic mice produces an aberrant hair phenotype. J. Cell Sci..

[101] Powell B., Walker S., Bawden C., Sivaprasad A., Rogers G. (1994). Transgenic sheep and wool growth: Possibilities and current status. Reprod. Fertil. Dev..

[102] Bawden C.S., Powell B.C., Walker S.K., Rogers G.E. (1998). Expression of a wool intermediate filament keratin transgene in sheep fibre alters structure. Transgenic Res..

[103] Bawden C., Penno N., Walker S., Hynd P., Rogers G. (2000). Genetic manipulation to modify wool properties and fibre growth rates. Proc. New Zealand Soc. Anim. Prod..

[104] Damak S., Su H.Y., Jay N.P., Bullock D.W. (1996). Improved wool production in transgenic sheep expressing insulin-like growth factor 1. Biotechnology.

[105] Su H.Y., Jay N., Gourley T., Kay G., Damak S. (1998). Wool production in transgenic sheep: Results from first-generation adults and second-generation lambs. Anim. Biotech..

[106] Adams N., Briegel J. (2005). Multiple effects of an additional growth hormone gene in adult sheep. J. Anim. Sci..

[107] Andl T., Reddy S.T., Gaddapara T., Millar S.E. (2002). WNT signals are required for the initiation of hair follicle development. Dev. Cell.

[108] Celso C.L., Prowse D.M., Watt F.M. (2004). Transient activation of β-catenin signalling in adult mouse epidermis is sufficient to induce new hair follicles but continuous activation is required to maintain hair follicle tumours. Development.

[109] Mou C., Jackson B., Schneider P., Overbeek P.A., Headon D.J. (2006). Generation of the primary hair follicle pattern. Proc. Natl. Acad. Sci. USA.

[110] Sick S., Reinker S., Timmer J., Schlake T. (2006). WNT and DKK determine hair follicle spacing through a reaction-diffusion mechanism. Science.

[111] Li W.R., Liu C.X., Zhang X.M., Chen L., Peng X.R., He S.G., Lin J.P., Han B., Wang L.Q., Huang J.C. (2017). CRISPR/Cas9-mediated loss of FGF5 function increases wool staple length in sheep. FEBS J..

[112] He D., Chen L., Luo F., Zhou H., Wang J., Zhang Q., Lu T., Wu S., Hickford J.G., Tao J. (2021). Differentially phosphorylated proteins in the crimped and straight wool of Chinese Tan sheep. J. Proteomics.

[113] Soller M. (1994). Marker assisted selection—An overview. Anim. Biotech..

[114] Gutierrez-Reinoso M.A., Aponte P.M., Garcia-Herreros M. (2021). Genomic analysis, progress and future perspectives in dairy cattle selection: A review. Animals.

[115] Chakraborty D., Sharma N., Kour S., Sodhi S.S., Gupta M.K., Lee S.J., Son Y.O. (2022). Applications of omics technology for livestock selection and improvement. Front. Genet..

[116] Saravanan K.A., Panigrahi M., Kumar H., Bhushan B., Dutt T., Mishra B.P. (2021). Genome-wide analysis of genetic diversity and selection signatures in three Indian sheep breeds. Livest. Sci..

[117] Sunnucks P., Wilson A.C., Beheregaray L.B., Zenger K., French J., Taylor A.C. (2000). SSCP is not so difficult: The application and utility of single-stranded conformation polymorphism in evolutionary biology and molecular ecology. Mol. Ecol..

[118] Sheep Genetics, University of New England, Armidale NSW. https://www.sheepgenetics.org.au/about-us/.

[119] Lincoln University Gene-Marker Laboratory, Canterbury, New Zealand. https://research.lincoln.ac.nz/testing-analytical-services/gene-marker-lab.

[120] National Research Council (2008). Changes in the Sheep Industry in the United States: Making the Transition from Tradition.

[121] Wuliji T., Dodds K., Land J., Andrews R., Turner P. (2001). Selection for ultrafine Merino sheep in New Zealand: Heritability, phenotypic and genetic correlations of live weight, fleece weight and wool characteristics in yearlings. Anim. Sci..

[122] Safari E., Fogarty N., Gilmour A., Atkins K., Mortimer S., Swan A., Brien F., Greeff J., Van Der Werf J. (2007). Genetic correlations among and between wool, growth and reproduction traits in Merino sheep. J. Anim. Breed. Genet..

[123] Huisman A., Brown D. (2009). Genetic parameters for bodyweight, wool, and disease resistance and reproduction traits in Merino sheep. 3. Genetic relationships between ultrasound scan traits and other traits. Anim. Prod. Sci..

[124] Mortimer S., Taylor P., Atkins K., Pope C. (2006). The Trangie QPLU $ selection lines: Responses in clean fleece weight and fibre diameter on completion of ten rounds of selection. Trangie QPLUS Merinos, Proceedings of the Trangie QPLUS Open Day, Trangie Agricultural Research Centre, Trangie, Australia, 11 May 2006.

[125] Ramos Z., Blair H.T., De Barbieri I., Ciappesoni G., Montossi F., Kenyon P.R. (2021). Productivity and reproductive performance of mixed-age ewes across 20 years of selection for ultrafine wool in Uruguay. Agriculture.

[126] Gong H., Zhou H., Hodge S., Dyer J.M., Hickford J.G. (2015). Association of wool traits with variation in the ovine KAP1-2 gene in Merino cross lambs. Small Rumin. Res..

[127] Mortimer S., Hatcher S., Fogarty N., van der Werf J., Brown D., Swan A., Jacob R., Geesink G., Hopkins D., Edwards J.H. (2017). Genetic correlations between wool traits and carcass traits in Merino sheep. J. Anim. Sci..

[128] Mortimer S., Hatcher S., Fogarty N., Van Der Werf J., Brown D., Swan A., Greeff J., Refshauge G., Edwards J.H., Gaunt G. (2017). Genetic parameters for wool traits, live weight, and ultrasound carcass traits in Merino sheep. J. Anim. Sci..

[129] Hanford K.J., Van Vleck L.D., Snowder G. (2002). Estimates of genetic parameters and genetic change for reproduction, weight, and wool characteristics of Columbia sheep. J. Anim. Sci..

[130] Hanford K.J., Van Vleck L.D., Snowder G. (2003). Estimates of genetic parameters and genetic change for reproduction, weight, and wool characteristics of Targhee sheep. J. Anim. Sci..

[131] Mortimer S., Hatcher S., Fogarty N., Van Der Werf J., Brown D., Swan A., Jacob R., Geesink G., Hopkins D., Edwards J.H. (2017). Genetic correlations between wool traits and meat quality traits in Merino sheep. J. Anim. Sci..

[132] Sheep Central (2023). Research Shows Good Eating Quality and Wool is Possible. https://www.sheepcentral.com/research-shows-good-eating-quality-and-wool-is-possible/.

